# Clindamycin as an Alternative Option in Optimizing Periodontal Therapy

**DOI:** 10.3390/antibiotics10070814

**Published:** 2021-07-04

**Authors:** Ionut Luchian, Ancuta Goriuc, Maria Alexandra Martu, Mihai Covasa

**Affiliations:** 1 Department of Periodontology, Faculty of Dental Medicine, “Grigore T. Popa” University of Medicine and Pharmacy, 16 Universității Street, 700115 Iași, Romania; Ionut.luchian@umfiasi.ro; 2 Department of Biochemistry, Faculty of Dental Medicine, “Grigore T. Popa” University of Medicine and Pharmacy, 16 Universității Street, 700115 Iași, Romania; 3 Department of Health and Human Development, University “Stefan cel Mare” Suceava, 13 Universității Street, 720229 Suceava, Romania; mcovasa@usm.ro; 4 Department of Basic Medical Sciences, College of Osteopathic Medicine, Western University of Health Sciences, 309E Second Street, Pomona, CA 91766, USA

**Keywords:** clindamycin, periodontitis, periodontal disease, oral biofilm, antibiotic therapy

## Abstract

Periodontal disease is an oral infectious and inflammatory disease caused by microorganisms that determine the host-mediated destruction of soft and hard periodontal tissues, which ultimately leads to tooth loss. Periodontitis affects a large part of the population, with various degrees of severity. Treatment consists of etiologic therapy: the removal of biofilm through mechanical debridement plus microbial elimination by supplementary measures. Antibiotic administration, either systemically or through local delivery, has been shown to improve clinical outcomes after mechanical periodontal treatment. Clindamycin is a lincosamide with a broad spectrum, being active against aerobic, anaerobic, and β-lactamase-producing bacteria. This antibiotic offers several advantages and some disadvantages and has been used in periodontal treatment both systemically and locally with various degrees of success. Among the properties that recommend it for periodontal treatment is the bacteriostatic effect, the inhibition of bacterial proteins synthesis, the enhancement of neutrophil chemotaxis, phagocytosis and the oxidative burst–oxidative stress storm. Furthermore, it is easily absorbed at the level of oral tissues in a considerable amount. This substantial tissue penetration, especially inside the bone, is synergistic with a stimulating effect on the host immune system. The aim of this review is to explore the applicability of this antibiotic agent and to evaluate its antimicrobial potential and limitations at the level of the oral biofilm associated with periodontal disease.

## 1. Introduction

The optimal antibiotic used in periodontal diseases should be bactericidal at therapeutic doses, have antimicrobial activity against a variable spectrum of pathogens specific to periodontal infections, should induce a minimal bacterial resistance, have good tissue penetration and be well tolerated [1,2]. Clindamycin is an antibiotic originally derived from lincomycin with a wide range of actions, with a high activity against Gram-positive aerobic bacteria and a broad range of anaerobic bacteria, among which are pathogens that produce beta-lactamase. In vitro and in vivo studies have shown that this drug reaches a high concentration at the site of infection, reducing the virulence of bacteria and increasing the phagocytic activity of host lymphocytes. Orally administered clindamycin is absorbed very quickly and efficiently, and its concentration remains at an optimal level for inhibiting microbial growth for at least six hours [3], proving to be highly effective, and capable of penetrating the supporting periodontium [4]. It can also be administered intravenously and has a remarkable tissue distribution and increased efficiency for *S. aureus* infections [5]. Furthermore, clindamycin has been suggested to exert immunomodulatory properties due to the suppression of proinflammatory cytokine release and its effects on phagocyte function, which is considered superior to dexamethasone with regard to *Porphyromonas gingivalis* [6,7]. Finally, clindamycin induces morphological changes at the surface of bacteria to facilitate their destruction and stimulates chemotaxis, thus inducing the mobilization of polymorphonuclear leukocytes at the site of infection and bacteria phagocytosis [8]. Clindamycin has a relatively short half-life and therefore requires administration every six hours to ensure adequate antibiotic concentrations. This review highlights the use of clindamycin as an alternative option in the treatment of periodontal disease within the context of currently available antibiotic and antimicrobial therapies and their route of delivery.

## 2. Clindamycin and Its Mechanisms of Action

Clindamycin exerts its bacteriostatic effect by inhibiting the synthesis of microbial proteins by binding to its RNA, the 50S subunit of the bacterial ribosome [9]. In addition to the direct antibacterial effect through ribosomal units targeting, clindamycin has a number of unique pharmacological characteristics that increase its clinical efficacy (Table 1). For example, clindamycin is the only antibiotic that reduces the adhesion of bacteria to epithelial cells on the mucosal surface by inhibiting the expression of virulence factors. This antibiotic inhibits the production of M proteins by group A β-hemolytic streptococci, thus blocking the formation of capsules by facultative species of Gram-positive streptococci. It also inhibits the bacterial proteins, enzymes, cytokines and toxins, such as those produced by *Clostridium* and *S. aureus* species [8]. Its capacity for cellular accumulation in neutrophil organelle due to the nucleoside transport system and the intracellular killing of bacteria is due to a proposed synergistic effect in the oxidative eradication mechanisms of clindamycin and neutrophils [10].

Furthermore, clindamycin suppresses the release of proinflammatory cytokines, such as TNF-α and IL-1β, that cause further destruction to the periodontal tissues when released in excess by bacteria and neighboring cells. Thus, the reduced production of TNF-α and chemokine CXCL-1 is another mechanism mediating the clindamycin inhibitory effect on inflammatory conditions such as periodontitis [6].

Finally, clindamycin acts by enhancing the functional capacity of polymorphonuclear neutrophils (PMNs) and bacterial killing. PMNs are very important cells in the fight against bacterial infections, and the ability of some bacteria to induce periodontal disease depends on the invasion of PMN defense cells on the presence of dysfunctional neutrophils [11]. The elimination of invading pathogens by PMNs in the periodontal tissues is accomplished through increased phagocytosis, the production of reactive oxygen species, degranulation and neutrophil extracellular traps (NETs). NETs are nuclear chromatin fibers mixed with antimicrobial molecules such as histones and peptides in a web-like structure that the neutrophil releases after activation. They act as a barrier for pathogens, limiting their spread and generating increased antimicrobial protein concentrations [12]. This is important for antimicrobial therapy, since an antibiotic that diminishes the release of autoantigens while at the same time avoiding the impairment of mechanisms of innate immunity is ideal, especially considering the fact that NETs release is affected in periodontitis.

Previous studies have shown that even sub-inhibitory doses of clindamycin can lead to increased bacterial opsonization and phagocytosis. This is accomplished by disturbing the synthesis of bacterial proteins, causing changes to the surface of the cell wall, leading to a decrease in the adhesion of bacteria to the host cell. The drug can exert prolonged effects for some strains of bacteria, even after the end of treatment, an effect attributed to the persistence of the drug at the binding site of the ribosome [13]. Orally administered clindamycin is absorbed by the small intestine after being hydrolysed. It is then distributed to the tissues, except the meninges; therefore, it is not used in brain infections. Clindamycin is metabolized in the liver primarily by the Cytochrome P450 3A4 enzyme and CYP 3A5, which oxidize the antibiotic into clindamycin sulfoxide (primary metabolite) and N-desmethyl clindamycin, respectively [13].

## 3. Resistance to Clindamycin

Clindamycin belongs to MLS_B_ class (macrolide–lincosamide–streptogramin B) of antibiotics that are known for inducing resistance to pathogens which may increase the risk of clinical failure. There are several main mechanisms involved in macrolide resistance to staphylococci. These are macrolide efflux, controlled by the *msrA* gene, changing the binding site of the drug to the ribosome, controlled by *ermC* genes (erythromycin ribosome methylation) and the enzymatic inactivation of antibiotics. Previous studies have shown the involvement of four *ermA*, *ermB*, *ermC* and *ermF* genes frequently associated with MLS_B_ resistance [14]. *ErmA* and *ermC* genes are usually classes of staphylococcal genes, *ermB* class genes are found mainly in streptococci and enterococci, while *ermF* class genes are present in Bacteroides and other anaerobic bacteria. In addition to *ermB* genes, *ermTR* genes, which are considered a subset of the *ermA* class based on sequence homology, can be detected in β-hemolytic streptococci [14]. The fact that each class is relatively specific, but not strictly limited to a bacterial gene reflects the easy exchange of genes. These genes produce methylase, an enzyme that alters the target site on ribosomes, thus preventing antibiotic binding, leading to both constitutive and inducible resistance [15]. However, previous studies demonstrated that among several antibiotic options used in the treatment of periodontal disease, clindamycin was the most efficient, mainly due to its reduced bacterial resistance [15].

## 4. Clindamycin’s Adverse Effects

Notwithstanding the biological and pharmacological properties of clindamycin reviewed above, its administration should be considered against the backdrop of the reported side effects (Table 1). In general, clindamycin is considered a safe drug for most people, except for pregnant and breastfeeding women [5]. Among the most common side effects are diarrhea, nausea, loss of appetite, abdominal discomfort and increased risk of *Clostridium difficile* infection. Clostridium difficile-associated diarrhea (CDAD) due to clindamycin can range from mild diarrhea to fatal colitis, occasionally even two months after stopping the antibiotic treatment. An excessive increase in *C. difficile* from clindamycin use, together with the production of A and B toxins, may contribute to morbidity and mortality in these patients. This risk occurs when the balance of the bacterial flora in the intestine is significantly altered, and therefore, the use of probiotics is recommended to avoid the development of *C. difficile*. It should be noted, though, that *C. difficile* is not involved in periodontal disease pathogenesis and usually, the administration of clindamycin for periodontal disease is limited for a short period of time [16]. Other reported adverse reactions include eosinophilia, skin rash, oesophagitis, and in rare cases, rheumatoid arthritis, Stevens–Johnson syndrome and hypotension. Hematologically, increased serum transaminases, neutropenia, leukopenia, agranulocytosis and thrombocytopenic purpura have also been reported. In rare cases, renal changes such as proteinuria or azotemia may occur. All these adverse reactions should be considered and should be managed using a dose-reduction strategy. In conclusion, considering its associated risks, clindamycin should be used with caution and when less toxic antimicrobial agents are inefficient [17].

**Table 1:** Advantages and disadvantages of clindamycin use [9,13,17,18,19,20].

Advantages	Disadvantages
Can be administered in a multitude of formulations, both locally and systemically	Primary adverse effects of clindamycin with systemic administration are allergic reactions, pseudomembranous colitis, nausea, vomiting, and diarrhea
Efficiency is not affected by diet	Cannot be administered to patients with a history of pseudomembranous colitis or ulcerative colitis or to pregnant persons
Active against most aerobic Gram-positive, anaerobic Gram-positive and Gram-negative bacteria	Aerobic Gram-negative bacilli are usually resistant due to poor permeability of the cellular outer envelope
Active against most periodontopathogenic bacteria (*Actinomyces, Eubacterium, Bacteroides, Prevotella, Porphyromonas, Fusobacterium, Veillonella* spp.)	Involved in antibiotic-associated diarrhea due to *Clostridium difficile* overgrowth
Reduces adherence of bacteria to host cells, increases intracellular killing of susceptible organisms	Can cause taste disorders, oesophagitis and changes in hematological parameters
Good penetration inside supporting periodontium	Mechanisms of antibiotic resistance: bacterial cell impermeability, target site alteration, enzymatic alteration or destruction of the antibiotic, increased efflux
Does not block the proangiogenic activity, thus having positive effect in the overall regenerative outcome	Insufficiently researched in clinical trials regarding periodontal disease activity in comparison to other more popular antibiotic regimens

## 5. Antibiotics in Periodontal Disease Therapy

### 5.1. Systemic Administration of Antibiotics in Periodontal Therapy

Patients with advanced forms of periodontal disease have subgingival bacterial plaque with colonies of *Porphyromonas gingivalis* and *Actinobacillus actinomycetemcomitans* [21]. *P. gingivalis* is a bacterium associated with aggressive periodontitis capable of producing enzymes that cause the destruction of the supporting connective tissue. *Actinobacillus actinomycetemcomitans* is associated with localized juvenile periodontitis and is capable of producing a leukotoxin that can damage the PMNs [22]. In a comparative study in patients with aggressive periodontitis and localized juvenile periodontitis, Eick et al. showed that the use of clindamycin resulted in a significant increase in phagocytosis and intracellular elimination of *P. gingivalis* and *A. actinomycetemcomitans* in patients with aggressive periodontitis and localized juvenile periodontitis as a response to the inflammatory reaction [23]. The prolonged use of antibiotic was not associated with lactic acidosis, neuropathy or myelosuppression [24]. Considering the fact that phagocytosis of microbes by resident crevicular PMNs is the cornerstone in the foundation of periodontal diseases development, the addition of clindamycin in the arsenal of periodontal therapy measures might offer additional benefits in the treatment of periodontitis.

Successful periodontal disease treatment is predicated upon selecting the suitable antimicrobial agent, the correct dose, along with the most advantageous administration route. All of these factors have a major influence on the treatment outcome. Clinical studies have tested many antimicrobial agents, such as amoxicillin, metronidazole, clindamycin, spiramycin, azithromycin, tetracycline, doxycycline, and their effects have been assessed for the treatment of aggressive and chronic periodontitis in several systematic reviews and meta-analyses [25,26,27,28,29]. For example, the susceptibility profile of microorganisms present in the oral environment, both Gram-positive and Gram-negative aerobes isolated from intraoral odontogenic abscesses was 78.13% and 81.48% for amoxicillin and clavulanic acid, the standard regimen; however, for clindamycin, it was 96.43% and 80.00%, respectively. Gentamicin and ciprofloxacin had the highest susceptibility profile for Gram-negative aerobes as all strains were found to be sensitive [30].

A recent study compared clindamycin with minocycline in order to assess angiogenic potential and cytocompatibility at different antibiotic concentrations on dental pulp stem cells and human endothelial cells. Even at higher concentrations, clindamycin had a lower cytotoxicity when compared to minocycline. The authors observed that the latter impeded proangiogenic activity, whereas clindamycin did not, thus suggesting the replacement of minocycline with clindamycin for the beneficial effects in the overall regenerative outcome [20]. Further, when comparing systemic clindamycin with tetracycline and metronidazole treatment, both treatments increased Immunoglobulin-G levels in aggressive periodontitis subjects, highlighting an improved immune reaction in this condition [31]. Thus, clindamycin displays activities in acute and chronic inflammation and an immunomodulatory effect that is highly suggestive of a pharmacological capability outside the limits of its antibiotic property. Lastly, it should be noted that antibiotics have various effects besides their antimicrobial properties, and they vary according to the specific class of chosen antibiotic, concentration, means of administration and target cells. For example, amoxicillin was found to induce NET release, and gentamicin, azithromycin and chloramphenicol have an inhibitory effect, while clindamycin has a neutral effect [32,33].

In general, the consensus is that systemic antibiotics should be used as part of non-surgical therapy, and due to the risk of the appearance of bacterial resistance or potential side/adverse effects, this indication is restricted to severe, progressive or aggressive periodontal disease forms [28,29]. Currently, there is no consensus on whether one antibiotic is superior over another, as they all exert similar clinical parameter improvements. However, the benefits of using antibiotics for pocket reduction and clinical attachment gain are evident, especially in more severe, progressive forms of the disease.

When prescribing systemic antibiotics, one must reach the desired pocket concentration and, unfortunately, not all antimicrobial agents are able to reach that area in a sufficient therapeutic dose, mainly due to inadequate tissue penetration [34]. Another drawback is the development of microbial resistance; thus, for the majority of cases, systemic antibiotic administration is generally reserved for subjects with severe generalized periodontitis. The periodontal pocket, due to its anatomy, although variable, is a very good site for local delivery of the drug, whose distribution is also facilitated by the crevicular fluid. Drug delivery systems that mold into the particular pocket shape improve bioavailability by encompassing all pocket areas [35]. Therefore, when comparing systemic versus local delivery systems, the latter possess a few advantages, such as a targeted minimal and direct application at the infection site, dose and frequency of administration cutback, gastro-intestinal evasion and first pass metabolism, the enhanced compliance of patients and incorporation of additional non-systemically deliverable agents (e.g., chlorhexidine) [36].

### 5.2. Local Antibiotic Delivery in Periodontal Disease Treatment

The most important benefit of local slow-release delivery systems is the extended and controlled dispensation of the drug in the periodontal pocket. This, however, implies the preservation of the system for a sufficient amount of time to be effective, and this can be a challenge in clinical settings due to tooth mobility, the natural communication between the periodontal pocket and oral cavity and the presence of gingival fluid. Given that not all systems meet these standards, they must be sealed inside the pocket with some type of adhesive, which can cause further irritation to the area and must be removed according to the manufacturers’ indications [34].

An important factor in assessing the efficiency of a local delivery system is not only the planktonic forms of periodontopathogenic bacteria, but also the degree of biofilm inhibition. As such, a 11.5% minocycline lipid complex inhibited biofilm formation even 28 days post-delivery [37]. In addition, special considerations should apply in hard-to-treat conditions or conditions that are refractory to treatment. In these cases, such as smoking, adjunctive antimicrobial use is especially useful. For example, a meta-analysis study showed that, in chronic periodontitis smoker patients, adjunctive local use of antibiotics yielded better clinical outcomes than scaling and root planing (SRP) alone, especially for deep periodontal pockets. Interestingly, these additional improvements were not seen in SRP plus systemic antimicrobial use [38].

Ideally, a locally delivered system must be biocompatible, biodegradable and non-irritant, have a sustained, controlled and prolonged drug release and be easy to use and apply. Currently, the available forms of administration are gels, films, fibers, strips, micro and nanoparticles and irrigation systems [39]. The most frequent indication is for localized forms of periodontitis and persistent, nonresponding, recurrent pockets [40]. Several drug delivery vehicles have been developed and used with various degrees of success. For example, Kilicarslan et al. developed a clindamycin-loaded complex film for local use in periodontal therapy prepared with chitosan and alginate with positive in vitro drug content, release, and adhesion [41]. Similarly, a nanofiber mat was constructed of a nanofiber layer of chitosan/poly(ethylene) oxide with 30% ciprofloxacin and a 5% metronidazole poly(ε-caprolactone) nanofiber layer [42] or a thermoresponsive, mucoadhesive, syringeable gel with levofloxacin and metronidazole [43]. These models achieved a slow, controlled release of active ingredients in periodontal disease treatment that were effective on a diverse spectrum of pathogens. As stated above, the effective treatment of wounds can be compromised by microbial biofilms because of the formation of a protective barrier formed by the secreted extracellular polymeric matrix. To overcome such challenge, a clindamycin-loaded copolymer nanogel was developed that was capable of breaking down the extracellular polymer substances of Gram-positive biofilms, attaching electrostatically onto the incorporated bacterial cells and transferring the antimicrobial directly onto their cell walls [44].

One way to improve the antibacterial activity of an antibiotic is by altering their physicochemical properties using the adhesion of nanoparticles to the bacterial cell wall. Hasan et al. prepared clindamycin-loaded nanoparticles positively charged and non-charged clindamycin in order to investigate the effect of nanoparticles on bacteria adhesion in the treatment of infected wounds with methicillin-resistant *Staphylococcus aureus*. Both systems had a sustained drug release, but the clindamycin nanoparticles were able to bind to the bacteria surface, augmenting the bactericidal potency when compared to the non-charged specimen, increasing the healing and wound re-epithelialization [45]. Taken together, both experimental and clinical studies reveal the importance of, and the need for, developing new delivery systems in the field of periodontology.

### 5.3. The Role of Antibiotics in Periodontal Therapy of Diabetic Patients

Antibiotic treatment in the cases of periodontal disease in patients with systemic impairment should be performed in a targeted manner after the microbiological identification specific to the case, in terms of both quality and quantity. Microbiological testing using the RT-PCR method is essential in establishing the treatment plan, but there are a number of controversies regarding the optimal antibiotic regimen for the diabetic patient. Diabetes is one of the main factors associated with periodontal disease; therefore, the therapeutic approach in this case requires maximum attention to detail [46]. Patients with uncontrolled diabetes have a significantly higher risk of developing severe periodontal disease [47].

Amoxicillin and metronidazole are frequently used in periodontal therapy as an adjuvant following scaling and root planing (SRP). Due to the increased resistance to the penicillin class as well as due to the frequent side effects of this combination, antibiotic alternatives have been sought, especially in patients with associated systemic diseases. Some have strongly argued that the optimal periodontal therapeutic approach in diabetic patients is SRP in combination with the systemic administration of amoxicillin and metronidazole [48,49,50,51]. At the same time, Gomez-Sandoval points out that there are no studies comparing the effectiveness of the combination of amoxicillin and metronidazole with clindamycin in the periodontal therapeutic management of the diabetic patient [52]. He showed that a seven-day period of antibiotics administration was preferable in periodontal therapy, using standard doses, to the detriment of a 14-day period of administration, as this reduces side effects and minimizes costs [52]. Alternatively, in a meta-analysis study, Rovai et al. evaluated the effects of SRP plus locally delivered antimicrobial agents vs. SRP alone, in subjects with type I and type II diabetes mellitus and chronic periodontitis. The authors noted supplementary benefits for adjunctive local antibiotic administration when compared to SRP alone in probing depth reduction and clinical attachment level gain, particularly in deep sites and well-controlled subjects [53]. These findings are in line with those of Gomez-Sandoval et al. who showed that the administration of clindamycin for seven days to diabetic patients with periodontal disease has similar effects to a combination of amoxicillin and metronidazole in reducing probing depth, the bacterial plaque index and the degree of bleeding during probing [52]. Although the efficacy of amoxicillin and metronidazole has been demonstrated by multiple previous studies, clindamycin is a real and viable alternative in the treatment of periodontal infections, and should be further investigated as a potential substitute.

## 6. Antibiotics vs. Other Antimicrobial Periodontal Therapies

Dental specialists frequently overprescribe antibiotics for specific conditions in order to reduce the symptomatology and postpone the treatment. This approach leads to increasing bacterial resistance to antibiotics and requires the search for other therapeutic protocols that include alternative solutions for antibiotic administration. Antimicrobial photodynamic therapy (aPDT) and laser therapy became popular and effective solutions used in periodontal therapy. Thus, they may be an alternative, in certain cases, to the systemic administration of antibiotics with similar results.

### 6.1. Antibiotics vs. Antimicrobial Photodynamic Therapy (aPDT)

Due to the potential side effects of antibiotic administration and to avoid surgery as much as possible, researchers are evaluating alternate therapeutic options adjunctive to non-surgical periodontal treatment. Antimicrobial photodynamic therapy (aPDT) has been proposed as an adjunctive alternate therapy that can offer additional clinical and microbiological periodontal improvements, especially in severe periodontitis cases, even in patients with fixed dental bridges that have an affected periodontal status [54].

Antimicrobial photodynamic therapy implies the application of a photosensitizer that binds to the target microorganisms and is activated by a light of a specific wavelength, generating reactive oxygen species and singlet oxygen that are toxic to the target pathogens. Although there have been studies that reported aPDT use in periodontal therapy, the results have been controversial: the beneficial effects were observed especially in regard to bleeding reduction, but there are no clear long-term results [55].

Whether aPTD is more effective than antibiotics as an adjunct to scaling and root planning is still debatable [56]. In a meta-analysis study, Souza et al. compared aPDT with systemic antibiotic use as adjuncts to SRP, and found similar improvement in the clinical status for both proposed treatments [57]. A 2019 systematic review analyzed whether SRP plus antimicrobial photodynamic therapy exerted an improved clinical outcome in comparison to SRP plus systemic antibiotics in periodontal disease. Although there were significant improvements for both treatment modalities, the inter-group discrepancy was not statistically significant in a consistent manner, and further studies are necessary in order to state a definite conclusion [58].

Another study sought to compare the clinical efficacy of repeated aPDT with that of systemic antibiotic therapy as an alternative approach to SRP in treating periodontitis by assessing pocket probing depth (PPD), clinical attachment loss (CAL) and bleeding on probing (BOP). Antibiotic therapy offered a significant gain in CAL when compared to repeated aPDT in deep pockets; however, no difference between the two methods was observed in CAL < 5 mm. Thus, antibiotics are still considered to be the principal therapeutic tool in the treatment of severe periodontitis [59].

Patients enrolled in supportive periodontal therapy that still had persistent or recurrent pockets were treated with SRP alone or in combination with local doxycycline administration or photodynamic therapy to assess clinical (PPD, CAL, BOP, plaque score) and microbiological treatment outcome. All three treatments yielded statistically significant improvements of clinical parameters without intergroup differences; however, periodontopathogens were diminished only in adjunctive therapy groups, with a statistically significantly higher reduction in the doxycycline group. As such, the adjunctive use of local antibiotics may surpass SRP alone or with aPDT in the ability to eliminate certain periodontal pathogens [60]. On the other hand, other studies found no additional benefits of either local minocycline application or aPDT when compared to SRP alone regarding clinical and microbiological parameters in deep or shallow periodontal pockets (PPD ≥ 6 mm) [61,62]. Together, these studies show a degree of variability in the effects of aPTD that are dependent on specific clinical parameters. Whether or not the local delivery of clindamycin associated with aPDT can significantly improve the clinical periodontal parameters is not clear yet.

### 6.2. Antibiotics vs. Laser Therapy

Lasers (mainly Nd:YAG, Er:YAG, Er, Cr: YSGG, and diode lasers) have been used as an adjunctive measure in nonsurgical periodontal therapy due to their bacterial destruction capabilities, the inactivation of endotoxins, the decontamination of the root surface area and the soft-tissue wall adjacent to the periodontal pocket by promoting bacterial reduction [63]. The majority of studies suggest that when lasers are used as monotherapy or adjunctive to SRP, the end results may be greater than SRP alone and should be considered a valuable adjunctive therapy variant.

There are very few studies comparing the effect of laser therapy as an adjunct to SRP and local antibiotic administration. One such study assessed the effects of the Nd:YAG laser irradiation into periodontal pockets with or without the combination of local antibiotic application on clinical and microbiological parameters. The combined laser plus antibiotic approach resulted in the greatest improvements in clinical parameters and the greatest reductions in *P. gingivalis, T. forsythia* and *P. intermedia* bacterial counts, demonstrating a synergistic effect [64].

Laser therapy has been more frequently compared to antimicrobial photodynamic therapy, and there is still no consensus as to which is more effective. While there are studies that find no difference between the two adjunctive methods regarding clinical outcome [65], others, such as the one from Grzech-Leśniak et al., observed added benefits regarding the clinical periodontal and plaque indexes for the Er:YAG laser and similar improvements to aPDT in some periodontopathogenic bacteria (*Prevotella intermedia, A. actinomycetemcomitans* and *Peptostreptococcus micros*) when compared to SRP alone [66].

It was observed that laser irradiation with diode and doxycycline on cultured human periodontal ligament cells induced a significantly diminished secretion of the matrix metallopeptidase 8 (MMP-8), with a maximum cutback for doxycycline, while collagen I secretion was only stimulated by the latter [67]. This suggests a dampening of the destruction processes at the periodontal ligament cells for both therapies and an improved regenerative outcome for the antibiotic.

A systematic review and meta-analysis assessed improvements in the nonsurgical treatment of chronic periodontitis patients by SRP with or without the following adjunctive: subantimicrobial-dose doxycycline as a systemic host modulator, the administration of systemic antimicrobials, or locally applied antimicrobial agents (minocycline microspheres, doxycycline hyclate gel and chlorhexidine chips and lasers (diode, Nd:YAG, Er:YAG, Er, Cr: YSGG and photodynamic therapy). SRP alone offered an average of 0.5 mm CAL improvement and associations of SRP with adjuncts improvement ranged between 0.2 and 0.6 mm compared to SRP alone. Systemic doxycycline in a subantimicrobial dose, systemic antimicrobials, local chlorhexidine chips and local photodynamic therapy were considered to have the best outcomes from the analyzed adjunctive substances [68]. Although reducing the prescription of antibiotic doses is a common current protocol, further studies should evaluate if the local delivery of clindamycin in a laser-sensitive environment can improve the results of periodontal therapy.

## 7. Clindamycin and Implant Therapy

Implantology has been an important field of periodontology for many years. Unfortunately, many patients with periodontal disease eventually end up with implant therapy that provides significant comfort through a minimally invasive solution. Oral implants are a viable, predictable and minimally invasive solution for replacing missing teeth. Although this domain has evolved greatly in the last decade, bacterial biofilm can produce a pathological form similar to periodontitis, namely periimplantitis. Implants may be vulnerable to damage from periimplantitis, but the approach of dentists regarding the prevention of implant contamination vary depending on the level of training and the country in which they practice. A recent observational study shows that specialists who practice oral implantology prescribe antibiotics as a prevention treatment both before and after the actual intervention [69]. However, the appropriateness of this practice is not supported by solid scientific evidence. The most commonly prescribed antibiotic is amoxicillin, while clindamycin is most commonly prescribed to patients allergic to penicillin. In order to reduce the over-prescription of antibiotics in the context of implant therapy, it is necessary to introduce clear clinical protocols regarding the recommendation of perioperative antibiotic treatment [69].

Further research is needed to determine whether replacing amoxicillin with clindamycin may improve implant prognosis. Recent research suggests that a new technology, 3D-printed CLIN, allows the stratification of titanium orthopedic implants in a compound that allows the long-term release of clindamycin to prevent postoperative infections [70]. This technology has all the prerequisites to be successfully introduced in oral implants, minimizing the risk of post-operative bacterial contamination and reducing failure rates.

## 8. Conclusions

Clindamycin is a reliable antibiotic option in periodontal therapy, with an excellent antimicrobial effect on the oral biofilm. Both systemic and local administration proved to be very efficient, easy to administer and clindamycin produces similar results to the popular combination of amoxicillin and metronidazole. However, treatment with clindamycin should take into account its potential side effects as well as avoiding potential allergic risk. Thus, antibiotherapy in treating periodontal disease remains a valuable resource; however, the route of delivery as well as the combination of antibiotic treatment with other modalities, such as aPTD or laser, should be considered on an individual basis applying the principle of personalized medicine. Further work should focus on oral biofilm bacterial resistance to clindamycin in order to validate a potential superior efficiency compared to other antibiotic alternatives as adjunctive solutions for successful periodontal treatment.

## References

[1] Dajani A.S., Taubert K.A., Wilson W., Bolger A.F., Bayer A., Ferrieri P., Gewitz M.H., Shulman S.T., Nouri S., Newburger J.W. (1997). Prevention of bacterial endocarditis: Recommendations by the American Heart Association. Circulation.

[2] Ehrenfeld M., Zambrano D. (1997). Clindamycin in the treatment of dental infections. Clindamycin in the Treatment of Human Infections.

[3] Brook I., Lewis M.A.O., Sándor G.K.B., Jeffcoat M., Samaranayake L.P., Vera R.J. (2007). Clindamycin for the treatment of dental infections. Rev. ADM.

[4] Patil V., Mali R., Mali A. (2013). Systemic anti-microbial agents used in periodontal therapy. J. Ind. Soc. Periodontol..

[5] Adhikari R.P., Shrestha S., Barakoti A., Amatya R. (2017). Inducible clindamycin and methicillin resistant Staphylococcus aureus in a tertiary care hospital, Kathmandu, Nepal. BMC Infect. Dis..

[6] Rodrigues F.F., Morais M.I., Melo I.S., Augusto P.S., Dutra M.M., Costa S.O., Costa F.C., Goulart F.A., Braga A.V., Coelho M.M. (2020). Clindamycin inhibits nociceptive response by reducing tumor necrosis factor-α and CXCL-1 production and activating opioidergic mechanisms. Inflammopharmacology.

[7] Nemec A., Pavlica Z., Nemec-Svete A., Eržen D., Milutinović A., Petelin M. (2012). Aerosolized clindamycin is superior to aerosolized dexamethasone or clindamycin-dexamethasone combination in the treatment of severe *Porphyromonas gingivalis* aspiration pneumonia in an experimental murine model. Exp. Lung Res..

[8] Brook I., Lewis M.A., Sándor G.K., Jeffcoat M., Samaranayake L.P., Rojas J.V. (2005). Clindamycin in dentistry: More than just effective prophylaxis for endocarditis?. Oral Surg. Oral Med. Oral Pathol. Oral Radiol..

[9] Spížek J., Řezanka T. (2017). Lincosamides: Chemical structure, biosynthesis, mechanism of action, resistance, and applications. Biochem. Pharmacol..

[10] Bongers S., Hellebrekers P., Leenen L.P.H., Koenderman L., Hietbrink F. (2019). Intracellular Penetration and Effects of Antibiotics on Staphylococcus aureus Inside Human Neutrophils: A Comprehensive Review. Antibiotics.

[11] Rosales C., Uribe-Querol E. (2017). Neutrophil role in periodontal disease. Role of Neutrophils in Disease Pathogenesis.

[12] Magán-Fernández A., Rasheed Al-Bakri S.M., O’Valle F., Benavides-Reyes C., Abadía-Molina F., Mesa F. (2020). Neutrophil Extracellular Traps in Periodontitis. Cells.

[13] Murphy P.B., Bistas K.G., Le J.K. (2021). Clindamycin. StatPearls.

[14] Timsina R., Shrestha U., Singh A., Timalsina B. (2020). Inducible clindamycin resistance and erm genes in *Staphylococcus aureus* in school children in Kathmandu, Nepal. Future Sci. OA.

[15] Rams T.E., Degener J.E., Van Winkelhoff A.J. (2014). Antibiotic resistance in human chronic periodontitis microbiota. J. Periodontol..

[16] Nasiri M.J., Goudarzi M., Hajikhani B., Ghazi M., Goudarzi H., Pouriran R. (2018). Clostridioides (Clostridium) difficile infection in hospitalized patients with antibiotic-associated diarrhea: A systematic review and meta-analysis. Anaerobe.

[17] Sharon S. (2007). Castle. Clindamycin. xPharm: The Comprehensive Pharmacology Reference.

[18] Thornhill M.H., Dayer M.J., Durkin M.J., Lockhart P.B., Baddour L.M. (2019). Risk of adverse reactions to oral antibiotics prescribed by dentists. J. Dent. Res..

[19] Mark Donaldson B.S., Jason H.G. (2017). Is clindamycin dangerous?. Gen. Dent..

[20] Dubey N., Xu J., Zhang Z., Nör J.E., Bottino M.C. (2019). Comparative evaluation of the cytotoxic and angiogenic effects of minocycline and clindamycin: An in vitro study. J. Endod..

[21] Cutler C.W., Kalmar J.R., Genco C.A. (1995). Pathogenic strategies of the oral anaerobe, Porphyromonas gingivalis. Trends Microbiol..

[22] Ashkenazi M., White R.R., Dennison D.K. (1992). Neutrophil modulation by Actinobacillus actinomycetemcomitans II. Phagocytosis and development of respiratory burst. J. Periodontal. Res..

[23] Eick S., Pfister W., Fiedler D., Straube E. (2000). Clindamycin promotes phagocytosis and intracellular killing of periodontopathogenic bacteria by crevicular granulocytes: An in vitro study. J. Antimicrob. Chemoter..

[24] Martínez-Aguilar G., Hammerman W.A., Mason E.O., Kaplan S.L. (2003). Clindamycin treatment of invasive infections caused by community-acquired, methicillin-resistant and methicillin-susceptible Staphylococcus aureus in children. Pediatr. Infect. Dis. J..

[25] Pretzl B., Sälzer S., Ehmke B., Schlagenhauf U., Dannewitz B., Dommisch H., Eickholz P., Jockel-Schneider Y. (2019). Administration of systemic antibiotics during non-surgical periodontal therapy—A consensus report. Clin. Oral Investig..

[26] Nibali L., Koidou V.P., Hamborg T., Donos N. (2019). Empirical or microbiologically guided systemic antimicrobials as adjuncts to non-surgical periodontal therapy? A systematic review. J. Clin. Periodontol..

[27] Teughels W., Feres M., Oud V., Martín C., Matesanz P., Herrera D. (2020). Adjunctive effect of systemic antimicrobials in periodontitis therapy: A systematic review and meta-analysis. J. Clin. Periodontol..

[28] Keestra J.A., Grosjean I., Coucke W., Quirynen M., Teughels W. (2015). Non-surgical periodontal therapy with systemic antibiotics in patients with untreated chronic periodontitis: A systematic review and meta-analysis. J. Periodontal. Res..

[29] Rabelo C.C., Feres M., Gonçalves C., Figueiredo L.C., Faveri M., Tu Y.K., Chambrone L. (2015). Systemic antibiotics in the treatment of aggressive periodontitis. A systematic review and a Bayesian Network meta-analysis. J. Clin. Periodontol..

[30] Bogacz M., Morawiec T., Śmieszek-Wilczewska J., Janowska-Bogacz K., Bubiłek-Bogacz A., Rój R., Pinocy K., Mertas A. (2019). Evaluation of drug susceptibility of microorganisms in odontogenic inflammations and dental surgery procedures performed on an outpatient basis. BioMed Res. Int..

[31] Krismariono A. (2009). Immunoglobulin-G level on aggressive periodontitis patients treated with clindamycin. Dent. J. (Maj. Kedokt. Gigi).

[32] Bystrzycka W., Moskalik A., Sieczkowska S., Manda-Handzlik A., Demkow U., Ciepiela O. (2016). The effect of clindamycin and amoxicillin on neutrophil extracellular trap (NET) release. Cent. Eur. J. Immunol..

[33] Bystrzycka W., Manda-Handzlikm A., Sieczkowska S., Moskalik A., Demkow U., Ciepiela O. (2017). Azithromycin and chloramphenicol diminish neutrophil extracellular traps (NETs) release. Int. J. Mol. Sci..

[34] Joshi D., Garg T., Goyal A.K., Rath G. (2016). Advanced drug delivery approaches against periodontitis. Drug Deliv..

[35] Steinberg D., Friedman M. (2020). Sustained-release delivery of antimicrobial drugs for the treatment of periodontal diseases: Fantasy or already reality?. Periodontology 2000.

[36] Rajeshwari H.R., Dhamecha D., Jagwani S., Rao M., Jadhav K., Shaikh S., Puzhankara L., Jalalpure S. (2019). Local drug delivery systems in the management of periodontitis: A scientific review. J. Control. Release.

[37] Schmid J.-L., Kirchberg M., Sarembe S., Kiesow A., Sculean A., Mäder K., Buchholz M., Eick S. (2020). In Vitro Evaluation of Antimicrobial Activity of Minocycline Formulations for Topical Application in Periodontal Therapy. Pharmaceutics.

[38] Chambrone L., Vargas M., Arboleda S., Serna M., Guerrero M., de Sousa J., Lafaurie G.I. (2016). Efficacy of local and systemic antimicrobials in the non-surgical treatment of smokers with chronic periodontitis: A systematic review. J. Periodontol..

[39] Jepsen K., Jepsen S. (2016). Antibiotics/antimicrobials: Systemic and local administration in the therapy of mild to moderately advanced periodontitis. Periodontology 2000.

[40] Herrera D., Matesanz P., Martín C., Oud V., Feres M., Teughels W. (2020). Adjunctive effect of locally delivered antimicrobials in periodontitis therapy: A systematic review and meta-analysis. J. Clin. Periodontol..

[41] Kilicarslan M., Ilhan M., Inal O., Orhan K. (2018). Preparation and evaluation of clindamycin phosphate loaded chitosan/alginate polyelectrolyte complex film as mucoadhesive drug delivery system for periodontal therapy. Eur. J. Pharm. Sci..

[42] Zupančič Š., Casula L., Rijavec T., Lapanje A., Luštrik M., Fadda A.M., Kocbek P., Kristl J. (2019). Sustained release of antimicrobials from double-layer nanofiber mats for local treatment of periodontal disease, evaluated using a new micro flow-through apparatus. J. Control. Release.

[43] Bansal M., Mittal N., Yadav S.K., Khan G., Gupta P., Mishra B., Nath G. (2018). Periodontal thermoresponsive, mucoadhesive dual antimicrobial loaded in-situ gel for the treatment of periodontal disease: Preparation, in-vitro characterization and antimicrobial study. J. Oral Biol. Craniofac. Res..

[44] Weldrick P.J., San S., Paunov V.N. (2021). Advanced Alcalase-Coated Clindamycin-Loaded Carbopol Nanogels for Removal of Persistent Bacterial Biofilms. ACS Appl. Nano Mater..

[45] Hasan N., Cao J., Lee J., Hlaing S.P., Oshi M.A., Naeem M., Ki M.H., Lee B.L., Jung Y., Yoo J.W. (2019). Bacteria-targeted clindamycin loaded polymeric nanoparticles: Effect of surface charge on nanoparticle adhesion to MRSA, antibacterial activity, and wound healing. Pharmaceutics.

[46] Taylor J.J., Preshaw P.M., Lalla E. (2013). A review of the evidence for pathogenic mechanisms that may link periodontitis and diabetes. J. Clin. Periodontol..

[47] Tsai C., Hayes C., Taylor G.W. (2002). Glycemic control of type 2 diabetes and severe periodontal disease in the US adult population. Community Dent. Oral Epidemiol..

[48] Mestnik M.J., Feres M., Figueiredo L.C., Duarte P.M., Lira E.A.G., Faveri M. (2010). Short-Term benefits of the adjunctive use of metronidazole plus amoxicillin in the microbial profile and in the clinical parameters of subjects with generalized aggressive periodontitis. J. Clin. Periodontol..

[49] Silva M.P., Feres M., Oliveira Sirotto T.A., Silva Soares G.M., Velloso Mendes J.A., Faveri MFigueiredo L.C. (2011). Clinical and microbiological benefits of metronidazole alone or with amoxicillin as adjuncts in the treatment of chronic periodontitis: A randomized placebo-controlled clinical trial. J. Clin. Periodontol..

[50] Miranda T.S., Feres M., Perez-Chaparro P.J., Figueiredo L.C., Tamashiro N.S., Bastos MFDuarte P.M. (2014). Metronidazole and amoxicillin as adjuncts to scaling and root planing for the treatment of type 2 diabetic subjects with periodontitis: 1-year outcomes of a randomized placebo-controlled clinical trial. J. Clin. Periodontol..

[51] Tamashiro N.S., Duarte P.M., Miranda T.S., Maciel S.S., Figueiredo L.C., Faveri M., Feres M. (2016). Amoxicillin plus metronidazole therapy for patients with periodontitis and type 2 diabetes: A 2-year randomized controlled trial. J. Dent. Res..

[52] Gomez-Sandoval J.R., Robles-Cervantes J.A., Hernandez-Gonzales S.O., Espinel-Bermudez M.C., Mariaud-Schimidt R., Martinez-Rodriguez V., Morgado-Castillo K.C., Mercado-Sesma A.R. (2020). Efficacy of clindamycin compared with amoxicillin-metronidazole after a 7-day regimen in the treatment of periodontitis in patients with diabetes: A randomized clinical trial. BMJ Open Diabetes Res. Care.

[53] Rovai E.S., Souto M.L., Ganhito J.A., Holzhausen M., Chambrone L., Pannuti C.M. (2016). Efficacy of local antimicrobials in the non-surgical treatment of patients with periodontitis and diabetes: A systematic review. J. Periodontol..

[54] Mocanu R.C., Martu M.A., Luchian I., Sufaru I.G., Maftei G.A., Ioanid N., Martu S., Tatarciuc M. (2021). Microbiologic Profiles of Patients with Dental Prosthetic Treatment and Periodontitis before and after Photoactivation Therapy—Randomized Clinical Trial. Microorganisms.

[55] Biel M.A. (2015). Antimicrobial photodynamic therapy for treatment of biofilm-based infections. Biofilm Based Healthc. Assoc. Infect..

[56] Akram Z., Hyder T., Al-Hamoudi N., Binshabaib M.S., Alharthi S.S., Hanif A. (2017). Efficacy of photodynamic therapy versus antibiotics as an adjunct to scaling and root planing in the treatment of periodontitis: A systematic review and meta-analysis. Photodiagnosis Photodyn. Ther..

[57] Souza E.Q., da Rocha T.E., Toro L.F., Guiati I.Z., Ervolino E., Garcia V.G., Wainwright M., Theodoro L.H. (2020). Antimicrobial photodynamic therapy compared to systemic antibiotic therapy in non-surgical treatment of periodontitis: Systematic review and meta-analysis. Photodiagnosis Photodyn. Ther..

[58] Pal A., Paul S., Perry R., Puryer J. (2019). Is the Use of Antimicrobial Photodynamic Therapy or Systemic Antibiotics More Effective in Improving Periodontal Health When Used in Conjunction with Localised Non-Surgical Periodontal Therapy? A Systematic Review. Dent. J..

[59] Ai R., Nie M., Yang J., Deng D. (2021). Effects of Antibiotics Versus Repeated Applications of Photodynamic Therapy as an Adjunctive Treatment for Periodontitis: A Systematic Review and Meta-Analysis. Photobiomodul. Photomed. Laser Surg..

[60] Cosgarea R., Eick S., Batori-Andronescu I., Jepsen S., Arweiler N.B., Rößler R., Conrad T., Ramseier C.A., Sculean A. (2021). Clinical and Microbiological Evaluation of Local Doxycycline and Antimicrobial Photodynamic Therapy during Supportive Periodontal Therapy: A Randomized Clinical Trial. Antibiotics.

[61] Tabenski L., Moder D., Cieplik F., Schenke F., Hiller K.A., Buchalla W., Schmalz G., Christgau M. (2017). Antimicrobial photodynamic therapy vs. local minocycline in addition to non-surgical therapy of deep periodontal pockets: A controlled randomized clinical trial. Clin. Oral Investig..

[62] Hokari T., Morozumi T., Komatsu Y., Shimizu T., Yoshino T., Tanaka M., Tanaka Y., Nohno K., Kubota T., Yoshie H. (2018). Effects of antimicrobial photodynamic therapy and local administration of minocycline on clinical, microbiological, and inflammatory markers of periodontal pockets: A pilot study. Int. J. Dent..

[63] Jia L., Jia J., Xie M., Zhang X., Li T., Shi L., Shi H., Zhang X. (2020). Clinical attachment level gain of lasers in scaling and root planing of chronic periodontitis: A network meta-analysis of randomized controlled clinical trials. Lasers Med. Sci..

[64] Noguchi T., Sanaoka A., Fukuda M., Suzuki S., Aoki T. (2005). Combined effects of Nd: YAG laser irradiation with local antibiotic application into periodontal pockets. J. Int. Acad. Periodontol..

[65] Freire A.E., Carrera T.M., de Oliveira G.J., Pigossi S.C., Júnior N.V. (2020). Comparison between Antimicrobial Photodynamic Therapy and Low-level laser therapy on non-surgical periodontal treatment: A Clinical Study. Photodiagnosis Photodyn. Ther..

[66] Grzech-Leśniak K., Matys J., Dominiak M. (2018). Comparison of the clinical and microbiological effects of antibiotic therapy in periodontal pockets following laser treatment: An in vivo study. Adv. Clin. Exp. Med..

[67] Dehdashtizadeh A., Esnaashari N., Farhad S.Z., Ejeian F., Amini S. (2020). The effect of laser irradiation and doxycycline application on the production of matrix metalloproteinase-8 and collagen I from cultured human periodontal ligament cells. Dent. Res. J..

[68] Smiley C.J., Tracy S.L., Abt E., Michalowicz B.S., John M.T., Gunsolley J., Cobb C.M., Rossmann J., Harrel S.K., Forrest J.L. (2015). Systematic review and meta-analysis on the nonsurgical treatment of chronic periodontitis by means of scaling and root planing with or without adjuncts. J. Am. Dent. Assoc..

[69] Salgado-Peralvo A.O., Kewalramani N., Peña-Cardelles J.F., Mateos-Moreno M.V., Monsalve-Guil L., Jiménez-Guerra Á., Ortiz-García I., Velasco-Ortega E. (2021). Preventive Antibiotic Prescribing Habits among Professionals Dedicated to Oral Implantology: An Observational Study. Antibiotics.

[70] Maver T., Mastnak T., Mihelič M., Maver U., Finšgar M. (2021). Clindamycin-Based 3D-Printed and Electrospun Coatings for Treatment of Implant-Related Infections. Materials.

